# Preventing the re-establishment of malaria in Sri Lanka amidst the COVID-19 pandemic

**DOI:** 10.1186/s12936-020-03465-5

**Published:** 2020-11-02

**Authors:** Prasad Ranaweera, Rajitha Wickremasinghe, Kamini Mendis

**Affiliations:** 1 Anti Malaria Campaign, 555/5 Public Health Building, Narahenpita, Colombo, Sri Lanka; 2 Department of Public Health, Faculty of Medicine, University of Kelaniya, Thalagolla Road, P.O. Box 6, Ragama, 11010 Sri Lanka; 3 Faculty of Medicine, University of Colombo, Kynsey Road, Colombo, Sri Lanka

**Keywords:** Malaria and COVID-19, Prevention of re-establishment of malaria, Multi-sectoral health collaboration, Quarantine, Contact tracing

## Abstract

The COVID-19 pandemic has had a considerable impact on other health programmes in countries, including on malaria, and is currently under much discussion. As many countries are accelerating efforts to eliminate malaria or to prevent the re-establishment of malaria from recently eliminated countries, the COVID-19 pandemic has the potential to cause major interruptions to ongoing anti-malaria operations and risk jeopardizing the gains that have been made so far. Sri Lanka, having eliminated malaria in 2012, was certified by the World Health Organization as a malaria-free country in 2016 and now implements a rigorous programme to prevent its re-establishment owing to the high receptivity and vulnerability of the country to malaria. Sri Lanka has also dealt with the COVID-19 epidemic quite successfully limiting the cumulative number of infections and deaths through co-ordinated efforts between the health sector and other relevant sectors, namely the military, the Police Department, Departments of Airport and Aviation and Foreign Affairs, all of which have been deployed for the COVID-19 epidemic under the umbrella of a Presidential Task Force. The relevance of imported infections and the need for a multi-sectoral response are features common to both the control of the COVID-19 epidemic and the Prevention of Re-establishment (POR) programme for malaria. Sri Lanka’s malaria POR programme has, therefore, creatively integrated its activities with those of the COVID-19 control programme. Through highly coordinated operations the return to the country of Sri Lankan nationals stranded overseas by the COVID-19 pandemic, many from malaria endemic countries, are being monitored for malaria as well as COVID-19 in an integrated case surveillance system under quarantine conditions, to the success of both programmes. Twenty-three imported malaria cases were detected from February to October through 2773 microscopic blood examinations performed for malaria in quarantine centres, this number being not much different to the incidence of imported malaria during the same period last year. This experience highlights the importance of integrated case surveillance and the need for a highly coordinated multi-sectoral approach in dealing with emerging new infections. It also suggests that synergies between the COVID-19 epidemic control programme and other health programmes may be found and developed to the advantage of both.

## Background

The impact of the COVID-19 pandemic on control programmes for other diseases, particularly malaria has so far being considerable, and is under much discussion [1, 2]. In many situations, the restrictions on human movement imposed by the COVID-19 pandemic even after lockdowns have been eased, have impeded the implementation of malaria operations in the field. The COVID-19 pandemic may also deviate limited financial and human resources away from other health programmes, such as malaria. Thus, the COVID-19 pandemic has the potential to hamper ongoing malaria programmes whether they be control, elimination or prevention of re-establishment programmes from recently eliminated countries, thus jeopardizing some of the gains that have been made worldwide in malaria. Sri Lanka eliminated malaria in 2012, but remains at high risk of malaria re-introduction and, therefore, conducts a rigorous programme to prevent the re-establishment of malaria [3, 4]. There are commonalities between the malaria Prevention of Re-establishment (POR) programme and the control of COVID-19, both programmes being at risk from imported infections, and both requiring stringent case surveillance, testing and follow up. Furthermore both the POR of malaria programmes and COVID-19 programmes entail working across many diverse sectors beyond health. Here we describe how the POR programme for malaria in Sri Lanka has integrated its activities into a well co-ordinated COVID-19 programme and thus highlight how potential synergies between COVID-19 and other health programme can be developed to the benefit of both.

## Interactions and integration between the COVID-19 and malaria prevention of re-establishment programmes

Imported malaria infections pose a continuing threat of re-establishing malaria transmission in Sri Lanka owing to the high prevalence of mosquito vectors in the country [3]. The country has been kept free of indigenous transmission during the 8 years since elimination except for a single case of introduced malaria reported in 2018 [5]. Sri Lanka has, so far, also fared well in the COVID-19 epidemic. As of 16 October 2020 there have been 5244 COVID-19 cases as reported from 365,859 real-time PCR tests performed in the country, and only 13 deaths in a population of 21 million [6].

The COVID-19 response in Sri Lanka has been one of testing for SARS-CoV-2 infection, rigorous contact tracing and quarantining, combined with mandatory wearing of facemasks, social distancing, and strict personal hygiene, after the 2-month lockdown was eased. Sri Lanka’s health system comprises 1.004 physicians per 1000 people. Contact tracing and quarantining requires an enormous effort on the part of health workers and is a heavy burden of work on the health system, and this was shared equally between the Ministry of Health and the Police Department and the military whose expertise and resources were rapidly mobilized.

The strategies of surveillance, contact tracing and testing have also been the core components of the POR programme for malaria. Since 2012, imported malaria patients and their travel contacts have been actively searched out by the Anti Malaria Campaign (AMC) and the Regional Malaria Officers, tested and treated to prevent the onward spread of their infections [3, 7]. Despite these commonalities, the staff, resources and expertise of the malaria programme were not mobilized for the COVID-19 response in Sri Lanka to any significant extent. However, several senior public health policy makers who were involved in the planning and implementation of the COVID-19 response have spoken out that their malaria elimination experience served them extremely well in planning for the COVID-19 epidemic. More specifically, their past experiences with investigating a case of malaria at the local level to ascertain where mosquito transmission may have occurred by tracing the movements of the person, and screening those who might have been similarly exposed to malaria by blood examination, and thereafter taking action at the local level, such as by focal vector control to prevent further transmission were, they said, principles which they have adopted in the COVID-19 response. They also said that appreciating the value of public health workers at the ground level in case investigation and local malaria control, and the importance of mounting coordinated responses were experiences they gained from malaria. What has also been remarkable from the beginning of the COVID-19 epidemic was the very efficient networking and highly organized and well coordinated operations between units of the Ministry of Health and other sectors whose inputs have been critical to the success of the programme—the Ministries of Defense, Airport and Aviation, Foreign Affairs, and the Police Department, all of which came under the umbrella of a Presidential Task Force for COVID-19 control.

Interactions between the two diseases, malaria and COVID-19 were many: the closure of the airport except for the repatriation of Sri Lankan nationals who were stranded overseas, was expected to be a respite for the malaria programme because of reduced traffic from overseas and, therefore, could have reduced number of imported malaria entering the country, but this was not to be. Many Sri Lankan returnees from overseas were from malaria endemic countries—Africa, the Middle East and India, which placed the country at high risk of importing malaria infections.

The repatriation of Sri Lankan nationals from overseas during the COVID-19 pandemic entailed them being flown back by the national airline, testing at the airport, immediate dispatch to quarantine centres for 2 weeks followed by a supervised self-quarantine in their homes for a further 2 weeks. This programme was coordinated between the Departments of Airports and Aviation, the Ministry of Defense (the Army, Air force and Navy), Ministries of Foreign Affairs, and Health. When Sri Lankan nationals are being flown into the country in batches during the current COVID-19 epidemic the Anti Malaria Campaign (AMC) is being informed by the relevant units of the COVID-19 programme of all details ahead of their arrival, including which countries they are arriving from, and the location of their to-be quarantine centres. If they happen to come from a malaria endemic country the AMC and the Regional Malaria Officers ensure that they are screened for malaria regardless of symptoms, by microscopic blood examination, once while in quarantine at around day 10 when blood is being taken for COVID-19 testing. In the post-quarantine period, they are followed-up with blood screening for malaria by microscopy at 3, 6 and 12 months when they return to their homes and they are advised to report for malaria testing if they develop fever. If and when a patient develops fever whilst in quarantine they are tested promptly for malaria. Furthermore, if the quarantine centre is located in a previously malaria endemic area, where there is a risk of malaria being transmitted, an entomological survey is conducted around the quarantine centre, and if malaria vectors are found, a pre-emptive vector control programme is implemented promptly. Accordingly entomological surveys have been conducted in quarantine centres located in 18 of the 25 districts in the country. Of a total of 80 such surveys, 17 surveys captured *Anopheles culicifacies*, the primary vector of malaria, which required vector control measures to be taken. Forty-four and 46 of these surveys captured secondary Anopheline vectors of malaria and non-vector Anopheline species respectively.

The AMC had cultivated close working relationships with the military and the Police Departments in the past during the elimination and post-elimination phases because their members were at a high risk of acquiring malaria, either in Sri Lanka during the civil war, or later while overseas on UN peacekeeping missions [8, 9]. These collaborations served well during the COVID-19 epidemic in which the military and the Police have been playing a major role.

Since the beginning of the COVID-19 epidemic in Sri Lanka a significant number of returnees from overseas developed malaria infections while they were in quarantine for COVID-19. A total of 23 imported malaria cases were detected from February to October through 2773 microscopic blood examinations performed for malaria in quarantine centres, this number being not much different to the incidence of imported malaria during the same period last year (n = 34). However, returnees from overseas being confined to quarantine centres (for COVID-19) made it all the more easier for the Anti Malaria Campaign to locate, screen and treat them promptly for malaria.

## Conclusion

Although the expertise and resources of the AMC were not directly utilized for the COVID-19 response, the Sri Lankan example highlights the importance of a highly coordinated programme between multiple sectors and departments that were involved in the COVID-19 response and the malaria POR programme. It also demonstrates the importance of a sound public health infrastructure with adequate, trained field staff for contact tracing and testing which was the common factor for the success of both malaria elimination and prevention of re-establishment, and the effective COVID-19 response in Sri Lanka. Thus synergies, and not always disruptions may be found between seemingly competing health programmes such as, in this case, COVID-19 and malaria. Functional, effective and highly responsive public health programmes with grassroots level public health staff, which are central to surveillance, contract tracing and quarantining and a well coordinated multi-sectoral approach may be important in today’s world, which is threatened by emerging and re-emerging infectious diseases.

## Data Availability

Not applicable.

## Abbreviations

AMC: Anti malaria campaign
POR: Prevention of re-establishment
